# Sharing Perspectives: Inviting Playful Curiosity Into Museum Spaces Through a Performative Score

**DOI:** 10.3389/fpsyg.2022.825625

**Published:** 2022-06-09

**Authors:** Andreas Løppenthin, Dorte Bjerre Jensen, Cordula Vesper, Andreas Roepstorff, Joseph Dumit

**Affiliations:** ^1^Interacting Minds Centre, Aarhus University, Aarhus, Denmark; ^2^Science and Technology Studies, University of California, Davis, Davis, CA, United States

**Keywords:** play, white cube gallery space, score, contact improvisation, art/science, museum studies

## Abstract

We report on the performative score “Sharing Perspectives” from the art/science research collaboration, Experimenting, Experiencing, Reflecting. Sharing Perspectives (SP) is developed as a score, inspired by choreography and the postmodern dance form Contact Improvisation, to stage exploration and improvisation, exploring uncertainty, creativity, togetherness, and the relationship between bodies and between bodies and space and artworks. The SP score acts as an experiment in how a brief intervention may affect the way art exhibitions are experienced, exploring how deeper and more sensorial engagement with art may be facilitated, for the benefit of visitors, galleries and artists. Based on questionnaires and qualitative interviews with participants during the Olafur Eliasson exhibition “In Real Life” at the Tate Modern in London in November 2019, we explore how the SP score modulates a playful mode of being, enhancing the experience of a museum art exhibition as a space of transformation and reflection. We find that the SP score encourages curiosity, which allows participants to recognize their habits for art and instead experience art slowly, recognize their comfort zones and move past them. As the score enacts a sensorial and playful approach to the exploration of the exhibition, participants experience a breaking of boundaries between each other, toward the other visitors, as well as to the artworks and the space itself, prompting an experience of being part of the exhibit as a whole. We discuss how the SP score invites a slowness and curiosity that takes on characteristics of play, which can change the participants’ appreciation of an art space.

## Introduction: Playfulness, Improvisation, and Transformation

In this article, we explore how the concept of a “score”, found in performance art and Contact Improvisation (Bjerre Jensen, 2020), can be employed in a museum setting as a way of countering museum fatigue and visitor experiences of alienation in fine art exhibitions—often described as “the white cube problem” (Birkett, 2012; Smith et al., 2017).

The article builds upon the movement-score ‘‘Sharing Perspectives’’ (SP), performed in relation to the Olafur Eliasson exhibition ‘‘In Real Life’’ at the Tate Modern in London in November 2019, as part of the art/science research collaboration, Experimenting, Experiencing, Reflecting (EER).^1^ A total of 37 participants were invited to take part in the SP score, in which they explored and guided each other through the exhibition space. Through questionnaires and qualitative interviews with participants, we explore how the SP score modulated an attentive and playful mode of being, which transformed the experience of the art exhibition from a place of inhibition and reservation to a space of curiosity and reflection, creating the experience of new relations to attention, habit, other museum goers, the art works and the gallery space. Our findings highlight the SP score as a way of engendering playfulness as a sensibility of competence and creativity, which supports agency, confidence and curiosity in the engagement with art, and leads to discussions of how the SP score’s playful disruptions of behavioral inhibitions relates to understandings of play as a way of learning, found within the cognitive sciences (Andersen et al., 2022).

### Practice and Theory

The interdisciplinary theoretical backdrop for this article spans our collaboration between performance studies on improvisation, museum behavior studies, and anthropological and cognitive approaches to play and habit. In the following, we situate our experimentation with the SP score within discussions of the museum space highlighting the contemporary art gallery as a “white cube,” where certain behavior and experiences are expected. Following this, we unfold the methodology of “scoring,” suggesting how it may be used to address issues of museum fatigue and experiential inhibition often experienced in the white cube (O’Doherty, 1999). Finally, we describe the theoretical perspectives employed when analyzing and discussing the experiences of participants in the SP-score, reflecting upon the implications of bringing together these different theoretical perspectives.

#### White-Cube Behavior, “Slow-Art” and Playfulness

Through beauty and provocation, folds and inspirations, museums and fine art exhibitions have the potential to be transformative spaces that create new reflections and understandings (Latour, 2013, p. 240). However, in spite of the hard work and best intentions of curators and museum staff, these experiences of transformation and reflection are often left unfulfilled, which has been documented through studies of museum visitor behavior, indicating an experience of museum fatigue and a tendency for visitors to move through art spaces at high speed, spending only minutes or even seconds with the exhibited objects (Bourdieu et al., 1991; Bourdieu and Johnson, 1992; Bitgood, 2009; Smith et al., 2017, p. 35ff). Within museum and gallery studies, discussions of visitor alienation have often been linked to an understanding of the contemporary museum space as a “white cube”—large rooms with high ceilings, (usually white) windowless walls, and artificial lighting illuminating few selected artworks (O’Doherty, 1999; Birkett, 2012; Lorente, 2015). As Lorente has argued, these architectural developments of the modernist museum space assume an ideal museum visitor as someone who moves carefully through the space in silent reverence of the aura of the artspace (Lorente, 2015, p. 115). While this atmosphere creates focussed attention around the artworks, the sacredness of the space may also intimidate visitors, making them feel embarrassed or alienated (Birkett, 2012, p. 2ff). In a seminal essay, Brian O’Doherty popularized the term “the white cube” as a timeless limbo-space, where only a very particular type of behavior is expected; no laughing, no loud voices, no eating, no drinking, no lying down or sleeping, no singing, no dancing (O’Doherty, 1999, p. 10). To O’Doherty, the white cube makes bodies obsolete, or even an intrusion. In his characterization, the contemporary artspace is intended for eyes and minds, not bodies (O’Doherty, 1999, p. 15). Although O’Doherty’s essay was first published in the 1980’s, the white cube experiences that he highlights are still evident in museums and galleries across the globe (Cain, 2017). The default organization of gallery spaces continues to be (usually white) windowless rooms with controlled lighting, with artworks displayed at eye-level, ensuring that visitor movements are as subtle and easy as possible—no bending over or crouching down (Cain, 2017).

Thus, although the term “white cube” refers to a specific color of paint and shape of room, the essence of this space is rather an effort to make the space itself invisible, creating a sacred aura of the artworks from which the ideal visitor experiences silent, personal epiphanies (Lorente, 2015). However, the narrow scripts of engagement alienate and push away many visitors, creating museum fatigue and feelings of inferiority (Bourdieu et al., 1991; Bourdieu and Johnson, 1992; Bitgood, 2009; Smith et al., 2017). The anxiety of museum limbo has perhaps found its solution in visitors speeding through galleries, where they attempt to “complete” the museum through checklists and selfies (Smith et al., 2017). Figuring out how to slow people down is difficult, and efforts have been done so under the broad concept of “slow art.” Shari Tishman has created workshops in “Slow Looking” as a way of learning that is about “taking the time to carefully observe more than what meets the eye at first glance” (Tishman, 2018, p. 2). Another attempt at slowing down museum visitors is the international event “Slow Art Day,” which is described by the initiator Phill Terry as “an antidote to that stress. Slowing down helps us see art in a new way that energizes rather than demoralizes” (ARTDEX, 2021). In addition to simply slowing down, Terry continues, “Many people don’t know how to look at and love art and are disconnected from it.” As part of the slow art movement, the Tate Modern in London has developed a guide for slow looking, as a way to spend more time with the artworks by asking: what will happen if we spend more time looking in detail at a piece of art (Tate, 2021)? The idea with slow looking is that, if we really want to get to know a work of art, we need to dwell. Finally, Johan Idema, art entrepreneur and author of “How to Visit an Art Museum” argues that galleries need to “work harder” for people to enjoy slow art by introducing more comfortable seating, having experts at hand to answer questions, or encouraging visitors to share their experiences (Idema, 2014).

While the Sharing Perspectives score was developed independently of museum contexts, its intervention echoes the “slow art movement” as one intervention into the white cube problem. We explore SP as a performative score in which participants are guided into a different way of experiencing and playing with being in museum exhibition. With the SP score, we have invited museum visitors to experiment with playful ways of engaging with the gallery space, that not only include the totality of the space, but also allows for bodily and sensorial interaction, challenging a tendency for a decoupling of mind and body in the exploration of fine art (O’Doherty, 1999, p. 15). As we will show, participants not only slowed down, but also invented their own way of appreciating the art and the museum context and thereby becoming part of the exhibit, in surprising, creative, and playful ways.

#### Scores as Staged Improvisations

Sharing Perspectives was originally designed by artist Dorte Bjerre Jensen as part of her “practice as research” MFA thesis, engaging university students in exploring bodily and spatial relations (Bjerre Jensen, 2020). Creating Sharing Perspectives as a score for participatory and collective explorations, Bjerre Jensen has experimented with merging the awareness of bodily communication, attention and sensibilities, found in contact improvisation, with academic inquiry (Little, 2014; Bjerre Jensen, 2020). With the SP score at Tate Modern, we applied the score as an exploration of encounters between visitors and artworks within a museum space.^2^ The SP score takes its point of departure in the performing and choreographic arts, and the practice of Contact Improvisation (Burrows, 2005; Little, 2014; O’Connor, 2019; Bjerre Jensen, 2020). Here, the concept of the score is understood as a set of instructions for a particular action, which could be actors performing on a stage or musicians playing in a band or, as in our case, visitors to a museum (Burrows, 2010, p. 141). Within a choreographic improvisation practice, scores are strategies that are open. They are not intended as blueprints for particular events, rather, they should be understood as a set of gentle, guiding instructions that constitute frameworks for improvisation, as a way of playing where the rules are not designed to regulate, but rather to foster a space for exploration and inventiveness (Burrows, 2005; Dumit, 2017; O’Connor, 2019, p. 112). The SP score draws on this practice of staged improvisation as a tool to enact playful explorations of relationships between bodies and artworks in a gallery setting, drawing on the score as a way of investigating and questioning habituated ways of moving and connecting (O’Connor, 2019, p. 112).

#### Theoretical Perspectives—Ways of Playfully Engaging With Art Spaces

The perspectives on the contemporary gallery space described above frame our understanding of the context in which the SP score unfolded. These ideas intersect a variety of disciplines and approaches—from sociological theory on habitus and practice (Bourdieu et al., 1991; Bourdieu and Johnson, 1992), over controlled experiments and observations of the behavior of museum visitors (Birkett, 2012; Cain, 2017; Smith et al., 2017), to theories on art, the moving body (within art museums) and architecture (Gil, 2006; Latour, 2013; Charmatz, 2014; Lorente, 2015). Although these perspectives have differing approaches, they share an orientation toward developing an understanding of the sociality, behavior and norms at stake within the contemporary museum space. When we analyze the experiences of participants in the SP score, we use the understandings presented in the white cube literature to contextualize the perspectives of the participants and frame our analytical arguments.

In addition to the white cube literature presented above, we rely on perspectives from a broader scope of philosophical and scientific fields when unpacking the participant experience. In particular, we discuss the SP-score in relation to a variety of perspectives on play on playfulness. Firstly, we relate the experiences of the participants in the SP-score to micro-phenomenological investigations of play. In an experimental context, Katrin Heimann and Andreas Roepstorff have explored how playfulness may be seen as an attitude that can be modulated to create experiences of competence and creativity (Heimann and Roepstorff, 2018). They found that verbally encouraging participants to solve tasks with a “playful” attitude made them more open and exploratory in their approach (Heimann and Roepstorff, 2018, p. 6). With these perspectives, we link the particpants experiences in the SP-score to a large and interdisciplinary field concerned with understanding how and why humans play. Within a framework of cognitive science, Marc Andersen, Julian Kiverstein, Mark Miller, and Andreas Roepstorff have discussed how human beings through playful engagement deliberately seek out and create surprising situations for themselves (Andersen et al., 2022). In this, Andersen and colleagues claim that play can be seen as a type of informal experimentation, through which agents gain experience and learn about the world around them. Thus, the authors argue that the act of playing facilitates creativity and innovations, which at its core is about setting up frameworks for surprise (Andersen et al., 2022, p. 23ff). Here, the authors highlight the pretend-play of children as a way of modifying environments so that they may create surprises. According to the authors, this expectation of being surprised is what makes playing so intriguing—we want to play because we want to be surprised. Andersen et al. (2022) thus argue that the process of playing is a type of experimentation that generates surprises in the moment, which will help prepare actors to resolve surprises in the future. In this way, playing becomes a way of learning through experimentation, leading the authors to suggest that play feels good because it over time transforms an unpredictable reality into a predictable one (Andersen et al., 2022, p. 10,15).

While these perspectives from micro-phenomenology and cognitive science help us recognize structures of playfulness in the participant experiences, we turn to theoretical perspectives from performance research and new materialism to unfold the specificity of the playfulness enacted and experienced in the SP-score. Firstly, we relate the SP-score to José Gil’s studies of moving bodies, and how, as bodies move in space, “the space of the body” becomes experienceable (Gil, 2006, p. 21). With this concept, Gil is calling attention to how a moving body “creates” space in its wake. Rather than seeing the body and space as two separate entities, Gil proposes understanding the moving body in space as porous and permeable matter that can be configured in multiple ways, and has the property of simultaneously being in space and becoming space, experimenting at each moment with how the world is unfolding and entangling with their bodies (Gil, 2006, p. 28). Furthermore, we employ Jane Bennett’s theorization of the role played by innate matter in social interactions as a prism to create a deeper understanding of how the participants in the SP score experience material connections in the gallery space (Bennett, 2010). Working within political philosophy and new materialism, Bennett suggests that playful bodies may extend and entangle themselves with other materials because matter has a vibrancy which gives it the capacity to affect courses of action. Bennett calls for expanding our understanding of what might carry agential capacity to include not just subjects but objects otherwise considered as inanimate (Bennett, 2010, p. 9). Bennett thus argues for an ontology of assemblage, understood as networks of actants wherein the intentional human is decentered, and emphasis is put on the multiplicity of matters and the agential potential of their vibrancy, their “thing-power” (Bennett, 2010, p. 36).

Thus, perspectives from white-cube literature inform our understanding of the contemporary art space, and situates the analysis of participant experiences as an exploration of modes of challenging the white cube. With perspectives on playfulness from micro-phenomenology and cognitive science, we describe and discuss these challenges as a type of playing. And finally, the specificities of this type of playfulness is unpacked through theoretical perspectives from performance research and new materialism. The goal of bringing together these different theoretical perspectives from multiple disciplines is not to develop one coherent theory on art spaces. Rather, these perspectives serve as tools to better understand the ways in which the SP score relates to art spaces and white cube behavior.

## Materials and Methods

This section describes the research design of our study and is divided into three subsections that match the three steps of the research process. Firstly, we describe the design and procedure of the SP score as an activity which generated particular experiences for the participants. Secondly, we describe the methodological design of the study, explicating our data generating methods. Finally, we describe the analytical design of the study, our data processing procedure and consideration of the validity and generalizability of the study.

### Score Design

In the context of Olafur Eliasson’s exhibition “In real Life” at the Tate Modern in London, we invited a total of 37 museum visitors and Tate employees to take part in Sharing Perspectives. The participants were spread out across seven sessions over 2 days, with 4–6 participants in each session, and led by authors Dorte Bjerre Jensen and Joseph Dumit (one facilitator per session). Participants were recruited through the Tate administration and researcher network. Tate employees took part in the score during work hours, while museum visitors were offered access to the exhibition after the score as compensation for their participation. The SP score was divided into three sections: a collective arrival, an individual exploration of the exhibition space, and finally sharing of perspectives with a partner. The score lasted about 45 min, followed by 45 min of the interviews and questionnaire responses.

#### Part 1: Arriving

The first part of the SP score consisted of a practical introduction and a grounding exercise. For the SP score to generate a space of shared exploration, it was integral to us that participants were assured that everyone had heard the same instructions, creating a common basis for improvising and experimenting with moving through space. This shared introduction took the form of a collective arrival that was not merely about conveying information on what was going to happen, but also to create a shared state of being. Here we gathered participants close together in a small circle, guiding them into feeling their bodies and encouraged them to begin to “notice how they are noticing,” creating awareness of their body in the moment, and their bodies in space together. The following is an excerpt from this introductory guidance by Dorte Bjerre Jensen:

‘‘So, let’s all just get a little bit closer. If you feel comfortable, you can close your eyes (pause). Just sense that we are together here, right now. Notice your breathing (pause). There are noises around us, they’re a part of being here. And then allow yourself to sense your body within this space and notice that you’re noticing. Start to pay attention to your standing body and the act of standing together with other bodies standing (pause). There are constantly small adjustments going on because we have chosen to stand. Just notice that you’re breathing and that you have a body here in this space. Maybe you’re noticing your heartbeat, maybe you’re noticing the contact to the earth. See if you can allow yourself to stand and notice what’s going on without trying to fix anything. Right now, we’re touching upon a movement practice on the activity of standing called the small dance.^3^ It is the dance the standing body has with gravity.”

The above quote illustrates how we intended to create a shared arrival space, where participants were attuned to themselves, the space and each other. The grounding arrival lasted approximately 4 min and was about opening participants to a fuller awareness of their sensorium, drawing attention to the multiplicity of ways in which the gallery space may be experienced, nudging participants to drop their habitual ways of moving and engaging with an art exhibition. Through this shared arrival, we intended to create a sense of collectivity, emphasizing that participants were part of a group exploring the space together, while simultaneously attuning themselves to the variety of sensorial experiences possible.

#### Part 2: Exploring

Following the shared arrival, participants were asked to spend 20 min exploring a limited area in the exhibition space (three rooms) while staying in the steady, grounded mode created when arriving. Borrowing the phrase “to move at the speed of your attention” from contact improvisation practitioner Nita Little, we encouraged participants to let themselves be guided by their impulses, allowing themselves to be drawn through the museum (Little, 2014, p. 250). The following is an excerpt from this guidance by Dorte Bjerre Jensen:

‘‘Allow yourself to explore space not only with your eyes, but with your whole being. We have all tried at one point to lean into a wall waiting for someone or something, or sitting on different surfaces, or laying down. Allow yourself to do so and at the same time notice how your body is adapting, sculpting and/or pouring its weight into a surface that you are touching or leaning against (Dorte demonstrates by leaning into a wall), and how the surface is responding to you by its touch and or its support—whatever you touch, is touching you back!’’^4^

In this mode of being, we then asked participants to let themselves wander through the space and notice three positions in the space that they for whatever reason found interesting at that moment, and then remember that place and how they positioned their body within it. When exploring these spaces, we encouraged participants to engage with the position and the perspective it gave them, trying to notice all the different ways they were experiencing it, including, but not limited to, its visual expression, sound, temperature, architecture and texture.

#### Part 3: Sharing

After spending time exploring the space on their own at the speed of their attention, letting themselves become attuned to notice how they were noticing, participants were paired (17 groups of 2 and one group of 3) and asked to share their three chosen places in space (place, position and duration) with their partner(s). This part of the SP score took another 20 min. Closely attending to each other, the groups of participants transitioned from one position to another by composing their bodies in space and guiding each other through the gallery space, introducing their partner(s) to the positions and perspectives that they had been attracted to when exploring on their own.

We asked participants to refrain from verbal communication, prompting them to find other ways of communicating, emphasizing that this sharing should be a collective bodily exploration, and that they should attempt to physically get into their partners’ positions, for example by imitating the movements and positions as closely as they could. Thus, while part 1 of the SP score attuned participants to a certain sensibility and part 2 prompted participants to use this sensibility to explore the exhibition space, part 3 of the SP score was about guiding another person through movement, without speaking with them, and attempting to share physical and bodily perspectives with each other. With these three steps—arriving, exploring, sharing—we set up a framework from which participants could explore and improvise, using the notion of the performative score as a gentle set of instructions that may allow new experiences to occur, attuning participants to an awareness of their embodied engagement with the museum space (O’Connor, 2019, p. 113).

### Methods Design

While the score procedure described above functioned as a generator for participant experiences, we employed a combination of group interviews and individual questionnaire responses as data generating methods. Immediately after finishing part 3 of the score, participants were invited to discuss their experiences with their partner(s) and a researcher in the form of semi-structured interviews, focussed on reflecting on the process through the main themes: How did they make decisions about their own positions? Were the experiences different when they entered their partners’ positions? Was attention shaped/changed over the course of the event, by the individual exploration and through the sharing of perspectives? (see Supplementary Appendix A for the interview guide). Participants were interviewed together with their partner(s) from the Sharing part of the score, and were encouraged to engage in reflective conversation, giving them space to discuss and unfold their experiences and opinions together. Interviews were performed by authors Andreas Løppenthin, Andreas Roepstorff, and Joseph Dumit. The interviews took place in the museum lobby, and lasted between 20 and 30 min. Interviews were recorded, transcribed, and anonymised; all participants given pseudonyms. Informed consent to use participants’ data was gained through a verbal consent statement, recorded in the beginning of the interviews.

Following the interviews, participants were asked to fill out a brief anonymous questionnaire about the experience on tablets provided by the researchers (see Supplementary Appendix B for questionnaire). 35 of 37 participants responded to the questionnaire. The combination of these methods allowed us to generate data on the collective reflection and meaning making of the experience of the SP score through the group interviews and to gather relevant background variables and potential anonymous feedback through the questionnaire.

### Analytical Design

The analysis in this article is based primarily on qualitative group interviews and has been performed in three stages—firstly, an inductive close reading of the interview transcripts, secondly a thematic coding based upon this inductive reading, and finally an abductive exploration of the interview material, developed in conversation between themes inductively derived in stage one and two and theoretical perspectives (Meyer and Lunnay, 2013, p. 2). The interviews were analyzed using a grounded theory inspired approach to map out the most prevalent themes in the data material, from which quotes were selected and unfolded in an abductive movement between data and theory (Thomas, 2010; Charmaz, 2016).

The first stage of the analysis, the inductive close reading, had the purpose of exploring prevalent themes in the interviews, and resulted in a set of interview displays—tables summing responses to the interview guide (see Supplementary Appendix C for interview displays). This process presented the contours of 4 main themes: (1) becoming attuned to new ways of seeing and experiencing an art space, (2) the experience of artworks, exhibition space and visitors coming together as one, (3) norm breaking, (4) understanding the perspectives of others. Based on these close readings, we developed a set of thematic codes to explore the interview data further, encompassing the second stage of the analysis (see Supplementary Appendix D for code overview). This coding process further qualified the themes found in stage one and served as a tool for quote selection for the next stage of the analysis. In the third stage of the analytical process, we began to explore the interviews with theoretical perspectives from the white-cube literature presented above, as well as perspectives on new materialism, the moving body and playfulness. In this abductive process, the analytical themes presented in this article were developed, as we turned our interpretive attention to understanding the SP score as a form of play that challenges art spaces characterized by white-cube behavior. This has the obvious implication that other aspects of the participants’ experiences of the SP score—such as interpersonal connection and the sharing of perspectives—are not addressed in this article. Thus, we do not claim that the analysis presented here encompasses the totality of the participant experiences. Instead, we point out that important aspects of the participant experiences can be related to understandings of playfulness, and that this contributes to an ongoing conversation about the link between learning, play and norm transgression.

### Scope and Limitations

In the above, we have described the design behind the study presented in this article. It consists of the SP score as an experience generating activity, interviews and questionnaires as data generating methods, and a threefold exploration of the interview data, with an inductive point of departure, building the basis for an abductive analysis. The aim of the analysis is to describe how the SP score unfolded under the particular circumstances of the “In Real Life” exhibition at Tate Modern in November 2019, and to examine what participants’ experiences may suggest about norms in art spaces and how they may be challenged. Our aim is not to prove that the SP-score will have the same effects in other times and places, nor argue that our interpretation of the participant experiences is the only valid understanding. Reproducing the specific context for our iteration of the SP score is neither possible nor a relevant goal in itself, and the validity of this study does thus not lie in our findings being representative or typical. Instead, the validity of our study is to be found through providing what Gary Thomas has termed as *exemplary knowledge*, that is, insights that may be provided through close and rigorous examination of a specific case (Thomas, 2010, p. 578). By describing the design and procedure of both the score itself, the data generating methods and our analytical strategy, we strive to provide the necessary transparency for others to follow how we reach our conclusions, but just as importantly because we hope that others will be inspired to applying to concept of scoring in other contexts—be that art spaces, workplaces or somewhere else altogether.

## Results: Breaking the White Cube—Playing, Connecting, Creating

In the following, we focus on the way in which the SP score interacts with (and disrupts) participants’ museum behavior. Drawing on interview responses, we focus on the participants’ reports of novel ways of entering and exploring an art exhibition and the affective components of these experiences. To that end, we examine how the participants experienced the SP score, highlighting how its playful character helped break down scripts of inhibition, allowing more profound and transformative connections to occur both between participants and to the art exhibition. We begin the analysis by describing background characteristics of the 37 participants in the SP score, as well as highlighting how they described their experience through questionnaire responses. Following this, we turn to the assessment of the qualitative interviews wherein participants describe their experiences. This analysis is focused on three main themes: (1) embodied experiences of the art space as one total installation, (2) experiencing the SP-score as a way of becoming part of the exhibition, (3) ways in which the SP-score challenges participant comfort zones and transgresses perceived norms through play. The fourth theme regarding understanding the perspectives of others will be discussed in a separate paper as our aim here is to focus on experiencing art and the museum space, as well as how a playful approach counters the white cube problem.

### Participant Background and Prior Experience

**FIGURE 1:**
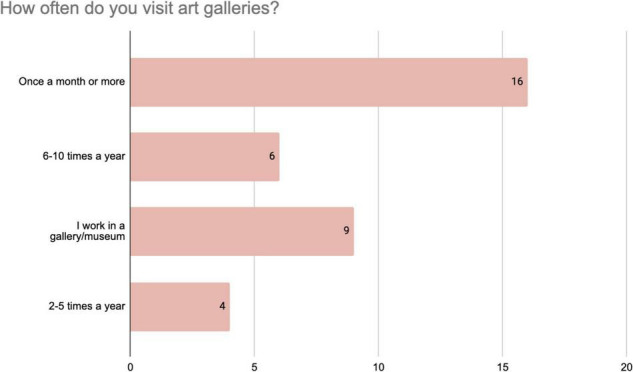
Distribution of responses from 35 participants to the questionnaire item about their visits to art galleries.

**FIGURE 2:**
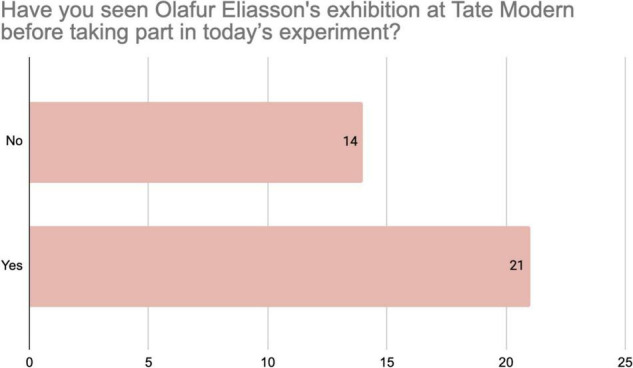
Distribution of responses from 35 participants to the questionnaire item about the “In Real Life” exhibition.

**FIGURE 3:**
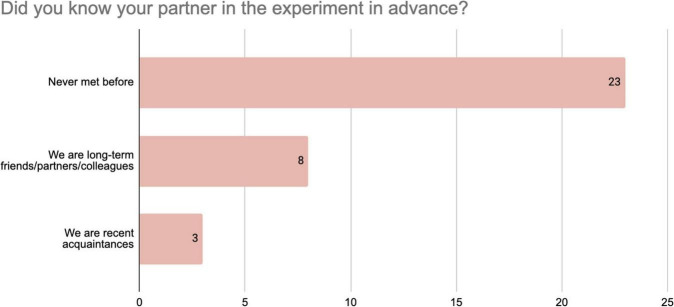
Distribution of responses from 35 participants to the questionnaire item about their relation to SP score partner.

Responses to the questionnaire provide insights into the background of the participants in the SP score. Responses to the question “How often do you visit art galleries?” (Figure 1) indicate that about half of the respondents (16 out 35) visit once a month or more, while 9 participants work in an art gallery. All the participants visit an art gallery at least twice a year. Figure 2 shows that the majority of the respondents (21 out of 35) had already seen the exhibition in which the SP score took place. Finally, Figure 3 shows that the vast majority of the respondents (23 out of 35) had never met before, while only 8 responded that they had a close relationship. In sum, these findings indicate the SP-score participants made up a group of art-knowers, who we can expect to be familiar with the conventions of art spaces, and that their shared explorations during the SP-score were generally made with an unfamiliar other person.

### Enjoying New Ways of Experiencing and Wanting More

When responding to the questionnaire questions regarding their experience of the SP score, *all* 35 participants responded that they would “Recommend a friend to participate.” These positive experiences were further elaborated in the comments section of the questionnaire, where participants were asked to “Explain your response” to the question of whether they would recommend the experience to a friend:

“I’ve never reflected much on the way I experience/move through exhibitions. The experiment suggested the idea of making this a conscious part of days out with a friend (not just galleries) and showed me that it can be rewarding.”

“I enjoyed it and felt encouraged to interact with the exhibition differently”

“It was a beautiful, fascinating experience which will stay with me, I felt it would be a great thing to do with a friend to understand them better”

**FIGURE 4:**
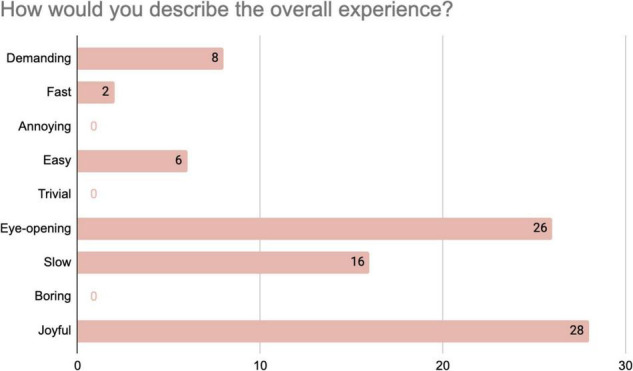
Distribution of responses from 35 participants to the questionnaire item about their overall experience. Note that participants could choose more than one word.

These expressions of enjoyment were confirmed when participants were asked to rate their experience on a scale from 1 to 10, as 22 out of 35 rated the experience 10, and none rated it below 7. When asked to choose words to describe their experience from a list of 9 adjectives^5^ (Figure 4), 28 out of 35 respondents chose the word “joyful” to describe their experience, 26 used “eye-opening,” while no one in our participant group chose to describe their experience as “annoying,” “trivial” or “boring.”

These responses suggest that the SP score does accord with the desire by museums for “slow art”. Not only did participants enjoy taking a leisurely time exploring the exhibits, but they also left in an encouraged and uplifted state, which differs from the museum fatigue often highlighted as a critique of the white cube (Bitgood, 2009) and most hour-long experiments. Rather than feeling drained, the SP score seemed to make participants want to come back for more and share their experience with their friends. In the following, we turn to unfold these experiences, relating them to understandings of the white cube and the concept of playfulness.

### Embodied Experiences of a Total Installation

The first theme of the participant experiences that we wish to highlight, centers upon experiences of becoming aware of how to connect to the art space through bodily explorations. As participants describe their exploration of the space in the interviews, many highlight how it led them to new and sensory ways of engaging with the artworks and the space. In this state of exploration, an immediate awareness of embodied connections seems to occur, as described in the following quote:

“My body told me: ‘You have to go down.’ ‘You have to understand where it [an artwork] is sitting.’ (…) I needed to feel dust on the ground, (…) you know, when it sits on this kind of plastic-gum black thing, and the dust was gathering around that. So, I needed to touch that, and I needed to actually have the dust on my fingertips.” (‘Billie’).

In this quote, Billie describes an inclination to touch the dust on the ground, and the plastic padding on the stands supporting an artwork. They^6^ emphasize an experience of their body ordering them to do something—that they “needed” to get in touch with the dust, describing an opening up toward bodily and sensorial explorations. In the quote above, Billie’s experience of the artwork is not described as an intellectual revelation on its meaning, nor is it articulated as a specific analysis of its shape or form. Rather, the artwork and the dust gathering around it creates a sensorial awe and wonder, which draws in Billie and profoundly touches them. In this way, we see Billie experiencing an awareness of their embodied relations to the artwork and space—their body “told” them how to explore the artwork, and they describe how it directed their understanding of it. In Billie’s description of this experience, we see an emergent alternative to the mind/body dualism often highlighted in literature on white cube behavior, where it is the “mind’s” understanding and appreciation of an artwork that is expected, and the rest of the “body” disregarded as an intrusion (O’Doherty, 1999, p. 15).

As the SP score asks Billie to play with their ways of exploring, their attention seems to be directed at a wider sensorium, in which verbal understanding is no longer privileged. This type of awareness of embodied exploration seems to be giving Billie access to a connection with the artspace. We are encouraged that Billie’s experiential language shares a theoretical kinship with new materialism where it is the *vibrancy* of the surrounding matters that draws them in and *affectively entangles* them (see Bennett, 2010 on vibrant matter and Hustak and Myers, 2012 for discussions on affective entanglements with materials). Thus, while the quote above illustrates a sensory and aesthetic attraction, it is not the artwork alone that is getting the attention. Rather, it is the relation between the artwork, the dust and the support stand that draws their attention—giving life to matters that are often rendered invisible by the hierarchies of the white cube. As the SP score orients participants toward their (already existing) sensory capacities, the culturally typical hierarchies of material importance seem to be disrupted. In the following quote, Pat describes what they call a “breaking of boundaries” between different matters, and how it pushed them toward a different way of experiencing the exhibition space:

“The whole experience helped me to break down those kinds of boundaries [between artworks, the space and visitors], and like the walls were really interesting to me. (…) I’ve stopped thinking about prioritizing artwork object over random bit of floor, random corner of ceiling. I just saw the entire thing as one total installation, with the people as well. And it’s just the—you could explore that all as one continuous surface.” (‘Pat’).

As Billie’s experience with dust and plastic padding, Pat’s experience of exploring the artspace through the SP score makes them shed ways of understanding hierarchies of matter within an art exhibition, and “random bits of floor” come to seem as significant as the artworks themselves. Rather than attempting to filter out other aspects than the artwork, Pat describes their experience as viewing the space as “one total installation”. This type of exploration differs from the understanding of the white cube as an invisible space for silent personal epiphanies (Lorente, 2015). Instead, Pat understands the totality of the exhibition space as ‘‘one continuous surface.’’ Compiling the experiences of Pat and Billie, it is striking that even in a space so minimalistic as the Tate Modern,^7^ the invisibility of the space may be challenged if visitors are encouraged to explore the space with a playful and bodily anchored approach. Although the only other things in the space apart from the artworks are the dust, the support stands and the walls, floors and ceilings, the totality of the space may still become apparent (and interesting) to the participants if they engage with it in this playful manner.

### From Viewer to Creator—Becoming Part of the Exhibition

The second analytical theme that we wish to highlight, relates to participant descriptions of connecting to the exhibition in ways that surprised them, even giving them a sense of agency in the creation of the artwork (experiences). In many cases, the SP score created attunements in the participants that drew their attention toward the space as a variety of entangled matters, creating what the participants describe as different and more profound connection to the whole exhibition:

“There’s a lot of the time that I feel I go to see things, and I’m looking so hard and I’m thinking so hard, that I’m actually quite detached from it and I don’t connect. Whereas doing it this way, as in we had that moment where we sort of, we built a team, we created a connection to each other, and then to ourselves, and then were taken straight into the exhibition. It felt very different to every other time I’ve done it [been to art exhibitions].” (‘Alex’).

While Billie and Pat quoted previously focus on how the SP score reshaped their attention, Alex highlights how the SP score relates to their typical experience of the art spaces. As they put it, they often find themselves “looking so hard” and “thinking so hard,” which leaves them detached from the artworks that they are really trying to connect with. This type of engagement—looking and thinking hard—resonates with the literature about the white cube described above, where the ideal visitor is someone who stays separate, moves through the space in silent reverence, taking in the aura of the artworks through their eyes and mind (O’Doherty, 1999, p. 15; Cain, 2017). To Alex, however, this intense looking and thinking is alienating, and the approach of the SP score offers them something quite different. To Alex, the SP score created a collective grounding, where they connected to themself and to the other score participants, which they describe as a change to their way of entering and engaging with the exhibition. It is building upon this group connection, that Alex has the experience of profoundly different ways of connecting with the artworks in the gallery:

“And being part of the exhibition, or feeling part of the exhibition was also quite a big part of it for me, because that immediately broke down the barriers of feeling disconnected from an artist whom I’m never met, and don’t know what he looks like, I just know his name. I really got involved in it and I attached myself to him, because I started to, you know, think about how I can understand when somebody makes work now, because it was happening to me. I felt like I’d made something” (‘Alex’).

Contrasting to their previous experiences of detachment, Alex describes that the SP score’s collective arrival and attunement enabled them to feel new connections. This is expressed through an experience of breaking down barriers toward an (on a personal level) unknown artist. Interestingly, what breaks down these barriers is not just an appreciation of the artworks, but the experience of being part of the exhibition, and contributing in the creation of the artworks, as they became aware of their own artistic role in experiencing art. As they explore the exhibition space through the attunement of the SP score, Alex gets the feeling that they ‘‘made something,’’ which attaches them to the artist as they co-create the artworks. While Olafur Eliasson’s work often invites participation, there is a profoundness in Alex’ feelings of attachment to the exhibition, which surprises them.^8^ Thus, when reading Alex’ description, we get a sense that they experience a certain vulnerability, where they “understand when someone makes work,” which indicates a feeling of shared responsibility that touches them. In this state of being, they are not merely a passive viewer of art, but instead an active participant in co-creating a transformative space of sensorial and perceptive exploration. Alex’s experiential-theoretical description mirrors the agentive materialist theories of, for example Latour who describes artworks’ potential for subjectivity transforming experiences (2013:240). Here we notice that scores like SP can enable participants to overcome museum-induced cultural scripts that prevent such potential from being realized.

### Challenging Comfort Zones

The two previous analytical themes presented draw attention to the participants’ description of the SP score as something that attunes them to engaging with an art exhibition, employing a wide spectrum of senses, which in turn engenders an awareness of the embodiedness inherent in exploring the exhibit. In this way, they depart from the kind of behavior that is usually expected in the white cube. To Billie, Pat and Alex quoted above, this opens up ways of perceiving that makes them feel closely connected to (or even part of) the exhibition. However, the SP score’s invitations to participants to move at the speed of their attention and expand their sensory attention, may also be a challenging or even uncomfortable procedure. We characterize this as the third theme of the analysis. In the following quote, Stevie describes their experience with a particular position that they took up when exploring the space:

“Stevie: I felt *really* exposed there. Because we were on view for—it was very clear, you could see people’s reactions coming in -

Micah: Even taking pictures.

Stevie: They were taking pictures of us. It was clear that we were—it was weird that we were standing there. So that intensity between your body and my body and the border between us, and this audience, I felt very exposed there. It felt very intimate.” (‘Stevie’ and ‘Micah’).

While we in previous quotes have seen participants that seem to lose themselves in their exploration of the space, Stevie finds themself in a position where they become self-conscious and nervous about how others perceive the way they are exploring the space. This makes them feel exposed, and as other visitors begin taking pictures of them, they become aware that they are breaking the norms of the space—what they are doing is “weird” and perhaps “too” intimate. Other participants also voice concerns of being weird, as Dylan who finds part of the SP score uncomfortable:

“It was uncomfortable actually. I’m not used to physically expressing myself in that way. I wouldn’t say it was a negative experience, but it certainly was out of my comfort zone. I’m a bit more—maybe you could tell from my earlier answer about being in a cocoon and not being touched or around people. You know, I like to keep to myself with it more, and keep tight, so to loosen and actually tangibly feel the wall was very different for me. I guess in one moment I was worried about whether people were looking at me weird. I heard people laughing behind me, and I was like “Do I look weird doing this?.”” (‘Dylan’).

For Dylan, the SP score asks them to express themselves in a way that they are not used to, and that puts them out of their comfort zone. By sticking out from the crowd they worry what other museum visitors might think of them, and whether they might laugh behind their back. The uncomfortableness in the moment makes them experience the norms of the white cube that they are simultaneously and actively choosing to break. This notion of being ‘‘weird’’ indicates to us that the implicit but quite specific norms of engagement in the white cube are not unproblematic to challenge. And in this sense, it is somewhat surprising that the concern for being ‘‘weird’’ is not more prevalent in the participants’ experiences.^9^ Here it is possible that breaking the norms of the white cube in the context of the SP score may come with rewards that outweighs the risks of being “weird”—as playing with the dust or turning attention to a “random bit of wall.” In the following, Pat unfolds their thoughts on this tension between the weird and the rewarding:

“I sort of feel like this [score] could be like the booze at a party. It basically breaks your inhibitions, and I think a lot of people that haven’t been raised in art are inhibited about how to act, and this is a way of smashing through that. Because you smash through to the other side, where you’re being *so* weird, as far as everybody else is concerned, but then to come back and just comfortably do whatever else you want with art, I think would be much easier than it had been before, because you’ve broken down all of these rules, essentially, about looking.” (‘Pat’).

To Pat, the “weirdness” of the SP score is not something that makes them uncomfortable. To them, it rather becomes a tool for consciously breaking down inhibitions of engaging with artworks. With this, Pat indicates that there are certain ways of acting within an exhibition space that can be difficult to inhabit for those that are not “raised” with them. By being “weird” they were able to “smash through to the other side” of these norms, where they could engage with their surroundings in norm transgressing ways, without worrying about social sanctions. Thus, to Pat, the weirdness of the SP score becomes empowering, helping them break free of the inhibitions of the white cube.

In conjunction, the above quotes reveal tensions at play, both within the SP score and the gallery space as such that are alienating and disconnecting for many. To these participants, the SP score is experienced as potentially disorienting, as something that balances on the edge of their comfort zone or makes them worry what others might think. For most, this disorientation offers gateways to an inspiring, profound and comfortable connection to the artspace. However, it is striking that the small changes to moving and exploring instantiated by the SP score are experienced with such a concern for diverting from the norm. This indicates that the “rules” (as Pat puts it above) of engaging with art spaces are experienced quite consciously and in an embodied way, as strong and restrictive, even for a group of people familiar with visiting art galleries (such as the SP-participants). In the context of fine art exhibitions, a concern for being the odd one might also be understood as a fear of not “understanding” the art. Thus, when Pat talks of inhibition as a result of not being raised in art, they are suggesting something akin to a painful experience of inferiority related to hierarchies of cultural capital—the gallery space can be a scary place if one has not been taught how to engage with it in the “right” way (Bourdieu et al., 1991; Bourdieu and Johnson, 1992, p. 35ff). The SP score thus calls attention to a tension within museums as to whether or not one is able to embrace the experience of following one’s curiosity even when it means being weird within the normativity of the space. What seems essential in decreasing this fear of sticking out is the experience of being part of a collective exploration authorized by the score facilitator. As is described in the following quote, the collective arrival and shared set of guidelines created a framework which authorized participants to be explorative and playful, as they were not just being “weird” on their own.

“I thought that the fact that we stood very close together [at the beginning] was really helpful, because it was the gentlest way of playing. It’s like ‘Here’s my personal space, and I’m just going to slightly overstep that first boundary.’ And I take that first step, and it turns out there’s nothing to be scared of. And at that point you’re just encouraged to play, gently breaking another boundary, one small step at a time until your confidence to do anything is kind of there.” (‘Pat’).

Describing how they were led into the SP score by the collective arrival, Pat unfolds how they slowly built up the confidence needed to step-by-step playfully engage with the space around them. In this way, the creation of a shared playful space allows them to challenge their own boundaries as well as the norms of the gallery space, to recognize that these norms are self-imposed. To Pat, the act of standing close to strangers in the SP score is the “gentlest way of playing,” which they describe as carefully challenging boundaries, learning that “there’s nothing to be scared of” and slowly building confidence.

As the ambivalence in the quotes above show, the structure of the SP score does not completely remove the tensions and concerns about misfitting in an art gallery. However, the interview data indicate that the SP score offers a possibility for participants to step into an explorative state of being, where they are less concerned with being right or wrong, and more concerned about engaging with the space around them.

## Discussion: Playful Curiosity, Competence, and Uncertainty

In the previous pages, we have explored how the participants’ experiences of Sharing Perspectives indicate that the score has a capability to transform engagement with art spaces, offering participants tools to challenge norms of how to act within the white cube of a museum context. As the SP score invites participants to experiment with their ways of moving through the space, it has the potential to transform the white cube from a space of alienating tacit rules and inhibition to a space of playful exploration.

In the following, we discuss how different disciplinary traditions of characterizing play can help us understand the SP score as a form of playfulness and discuss the implications this has for play as a form of cultural learning. These approaches include understanding micro-phenomenological research on the characteristics of playfulness, and particularly as a behavioral “mode” which it may be possible to encourage (Heimann and Roepstorff, 2018). We look also at performance research on sensory attunements through playful movement and attentional exploration leading to surprise (Gil, 2006). Finally, we discuss the implications of the SP score as a form of playing in relation to perspectives found in cognitive science literature that sees playing as a type of informal experimentation (Andersen et al., 2022). Here we argue that while playing, in a long-term perspective, may serve as a provider of experience that aids in reducing unpleasant surprises, the playfulness enacted in the SP-score works to attune participants to the myriad of ways an art space may be experienced, allowing them to create connections founded in surprises.

### Playfulness as an Attitude That Can Be Modulated

As previously described, micro-phenomenological research has linked play to exploration and curiosity (Heimann and Roepstorff, 2018). These experimental findings resonate with what we have described above about the experiences of the participants in the SP score, in particular that “freed from specific constraints and goals, participants seem to enter curiosity driven interaction with the material, which allows for an unknown outcome to occur” (Heimann and Roepstorff, 2018, p. 11). As the SP score encouraged participants to “move at the speed of their attention” and become attuned to a wider spectrum of their senses, they adopted a careful attentiveness in their engagement with the art space, which allowed for curiosity and creative connections. In the Heimann and Roepstorff experiment, participants were explicitly asked to perform a task in a playful manner (Heimann and Roepstorff, 2018, p. 4). In Sharing Perspectives, we rely on the practice of the score, inspired by choreography and contact improvisation, rather than a traditional scientific experimental prompt. Although we did not explicitly use the word “playful” in our framing of the score or toward participants (as Heimann and Roepstorff did), the explorative approach of the SP score had a clear playful feeling as described by participants, and in the implicit contrast to the seriousness with which art museums are often experienced. We therefore note that the SP score at Tate Modern indicates that scores may be used to create playful attitudes in the exploration of art exhibitions. Paraphrasing Heimann and Roepstorff, we may see the SP score as a way of modulating the participants into a particular playful mode of being, engendering competence, creativity and agency. This has important implications for the understanding of norms of gallery spaces, the so-called white cube behavior, and the hyper speed at which much art is viewed (O’Doherty, 1999; Smith et al., 2017). With the SP score, it appears to be possible to reframe the art gallery from a tense space with scripted ways of engaging with artworks, to a more open and exploratory space, characterized by slowness, playfulness, curiosity, and agency.

### Playing With the Senses—Attuning to Vibrancy

As we have argued through the analysis of participant experiences, the playfulness of the SP score relates to a heightened sensory awareness as participants become aware of the different possible ways of connecting with the surrounding space, and becoming entangled with the matters in the space—dust, walls, artworks, others. With its grounding in Contact Improvisation and its focus on starting from bodily attention and presence (Koteen and Stark Smith, 2021), we can understand the SP score as facilitating an opening of participants’ attention to the space-making of their bodies and the ways this spacing is part of the spacing of the museum and artworks within it. Thus, when participants report feeling as if they were part of the exhibition themselves, we are seeing how they become aware of the capability of their senses to playfully entangle with the spaces that they are in. In a performance studies phrasing, we suggest that this sensorial attunement is a type of attentional and bodily dancing, where participants begin to enter into a rhythm with the space and the various matters present in this space (Gil, 2006, p. 25), while also being able to notice that they are out of rhythm with the “white cube” dance of other museum visitors. The play involved in the SP score, is thus something that enhances the creative capabilities of participants, opening attention toward the multiplicity of impressions that their senses may give access to.

While José Gil’s perspectives may help us to become theoretically attentive to how the SP score creates awareness to the sensorial capacity of the spacetime of the body, Bennett’s (2010) account of the vitality and vibrancy of matter highlights material entanglements relevant to understanding how the participants connect to the space that they are in. In the case of the SP score, Bennett’s framework may be employed to understand the exhibition space as an assemblage of matters; of artworks, visitors and walls, and on an even more detailed level, of dust and airflows, sound waves, and particles of light. Through its instructive framing, the SP score attunes participants to explore these different matters and their vibrancy. Thus, it seems that in the process of slowing down and moving at the speed of their attention, participants open themselves to being affected, to being touched, by the complex assemblages of matter and space that make up the exhibition, an assemblage that they notice they are participating in making. While Bennett on an abstract level calls for the theorist to “admit a “playful element” into one’s thinking” (Bennett, 2010, p. 15), the SP score on a practical and sensorial level calls for participants to admit a playful element into engaging with an exhibition, including interactions between bodies, artworks and the space itself. In this way, we may understand the connections that the SP score creates as an attunement of the participants to the vibrant agency of the objects in the space and each other. In some cases, it attunes them to the animacy of the space itself, the play of light and shadow, the changing configurations of people in the rooms. The feeling of being “part of the exhibition,” as quoted above, can thus be seen as a way of connecting and involving oneself with the vibrancy of the matter making up the gallery space.

In these experiences, participants are perhaps echoing in their actions and reflections what choreographer Boris Charmatz described (about the art museum) as a nomadic, ephemeral and precarious assemblage of the mental and the spatial, as he argues that “the spirit of the place emerges before the place, that everything remains to be done, and that the daily life of this construction site makes room for every audacious idea and every eccentricity” (Charmatz, 2014, p. 47). In these ways, the doorway of permission to play introduced to participants by the SP-score—to play with their attention, to play with their positions, and to play with their postures—directly addresses the white cube problem presented in the beginning of this article. Participants either implicitly or explicitly become able to notice their social and habitual constrictions around “proper museum behavior” as inhibiting their ability to notice more about the art, the museum, and their appreciation of it. Through playing with their own act of attending, the participants’ time in the museum becomes a kind of “partial” event, that is part of the art without competing with it. This is facilitated because the SP score is a piece that exists only in the moments it is being unfolded by the participants. They are performing it. Along the way they report experiencing many other ways of seeing and being, as facilitated by the art and the setting. And they want to come back to the museum to see more!

With these perspectives, we have unfolded how the SP score acts as a facilitator for a type of playfulness that enables participants to experience connection with art spaces in explorative ways. We suggest that the SP score opens up this participatory vibrancy in art through the “play” of attention—as the participants notice their noticing within a frame of openness and playfulness, the space comes alive, able to be filled with more possibilities for noticing and for doing (for playing). By opening their sensory capabilities in a curiosity-filled way, participants allow themselves to be surprised, which in turn allows them to connect with the space, themselves and each other. This is a practice of empirical learning through leaning in and becoming affectively entangled with one’s object (Hustak and Myers, 2012).

### Playing to Learn and Learning to Play

The understanding of playful activities as informal experimentations that may yield new and surprising experiences, proposed by Andersen and colleagues (and unfolded above), resonates with our findings from the SP score (Andersen et al., 2022). As we have shown, the SP score sets the parameters for participants to explore and experiment with how they could experience the totality of the exhibition space, getting surprised and learning new things about themselves and the artspace. However, the type of playfulness instantiated by the SP score also seems to have other properties than the trial-and-error experimentation implied by the understanding of playfulness as informal experimentation that facilitates learning and improved prediction (Andersen et al., 2022, p. 9). When we assess participant experiences of the SP score, the playful attitudes encountered seem to be about getting lost rather than uncovering new paths. Thus, as unfolded with Gil and Bennett, the interviews we performed with participants suggest that the SP score creates an orientation toward curiosity, creativity and connectedness for the sake of those experiences in themselves, and as we have seen above, participants report how the SP score helps them break free of scripted ways of acting in a museum space that were felt to be constraining, helping them to find new and surprising connections.

Thus, the way participants in the SP score are playing is related to a search for openness and serendipity, rather than improving prediction and limiting uncertainty. While Andersen and colleagues highlight the role of playfulness in cognition and longer-term development, pointing out how playing is a way of learning, the SP score serves as an example of how playfulness in its situated nature may be a mode of open exploration—playfulness may become the point of a museum, learning to find new forms of uncertainty, curiosity, and joy as ends in themselves (see Graeber, 2014). As a score that facilitates new sensorial explorations of art spaces, the SP score turns the habituated (and predictable) ways of engaging with art spaces upside down, allowing for participants to stay with the unknown, emphasizing the openness and myriad of possible connections in the spacetime of the exhibition. In this situated context, the SP score is more about learning to play, than playing to learn.

Thus, the SP score creates a space of playfulness that transforms a predictable reality to an unpredictable one, uncomfortable and weird sometimes, but often leading to participants learning and even desiring to become “weirder”. Through a framework for improvisation, the SP score destabilizes the ways in which participants engage with art-exhibitions, by offering a glimpse into what could become the start of an un-learning of scripted ways of moving, making engagement with the white cube more open for surprises and intimate connections. In this space, participants become sensorially attuned to a wider scope of their experience. They thus experience interconnection not only between each other, but also to themselves, the space and the artworks. This suggests that inviting visitors to take part in collaborative performance artwork, such as a score, within a gallery space may enhance the experience of visiting an art exhibition, breaking down barriers and allowing participants to engage in exploring themselves and the space as one continuous surface. This creates spaces for transforming the visitor experience from passive to active, as participants become immersed in the exhibition, and experience themselves as taking part in an empathic togetherness with other matters—both the objects in the space, the art works, their partners, other museum visitors and the artist themself.

## Conclusion: Playing to (Un)Learn Ways of Engaging With Spaces

In this article, we have explored how the score “Sharing Perspectives” may work to enhance the transformative effects of art galleries and exhibitions. Developing the SP score based on contact improvisation and performance art, we have strived to set up a space for experimenting with movement in space, exploring how a collaborative performance piece such as Sharing Perspective may be able to create connections, engagements, and surprises between participants, space and art. The SP score thus allows for participants to experiment with their own as well as the boundaries of others and exhibition spaces, creating experiences that enable reflections upon their habituated way of moving in space and experiencing art. In this way, the SP score acts as an experiment in how a brief intervention may affect the way art exhibitions are experienced, exploring how deeper and more sensorial engagement with art may be facilitated, for the benefit of visitors, galleries and artists.

We find that by suggesting a playful attitude to the participants, it is possible to engender an experience of creativity and competence when exploring an exhibition space otherwise encoded with specific modes of engaging and understanding. In this way, the SP score facilitates a “slow” and sensorial approach to an exhibition, in which participants describe the breakdown of boundaries between themselves, the other visitors, the artworks and the space, prompting an experience of one total installation with a continuous surface.

Bringing together theoretical perspectives on moving bodies and the vibrancy of materiality, with research on playfulness, we argue that the SP score adds to cognitive science perspectives on the long-term explanations for playfulness, by exploring playfulness in a situated case (Gil, 2006; Bennett, 2010; Heimann and Roepstorff, 2018; Andersen et al., 2022). In contrast to highlighting playfulness as a form of experimentation, with the long-term purpose of transforming unpredictable worlds into predictable ones (Andersen et al., 2022), the playfulness experienced by participants in the SP score seems better described as a way of transforming the static scripts and inhibitions of art spaces into curious and creative explorations that are meaningfully experienced here-and-now. Rather than seeking to get better at predicting and “understanding” art spaces, the SP score offers participants a framework of playfulness from which they can stay with an uncertainty that engenders novel connections, understandings and perspectives, transforming a predictable world to a surprising and unpredictable one, that one may joyfully and curiously explore with others. Thus, the SP score offers participants an embodied attention that allows for playful ways to challenge the expectations and scripts of the highly coded white cube of an art gallery. Paradoxically, this may be an ultimate and transferable learning experience. This is at least suggested by an unsolicited email we received from a participant a few days after the SP score took place:

“I thoroughly enjoyed participating. And, to my delight and surprise, find that it has an impact now days after, as I register that I’m thinking of exchanges with friends differently, being more aware of what they might have meant, and how what I did or said could have been perceived. In short, being more aware.”

## Data Availability Statement

The raw data supporting the conclusions of this article will be made available by the authors, without undue reservation.

## Ethics Statement

Ethical review and approval was not required for the study on human participants in accordance with the local legislation and institutional requirements. Written informed consent for participation was not required for this study in accordance with the national legislation and the institutional requirements.

## Author Contributions

AL contributed to the primary analysis. JD and DBJ contributed to the primary analysis and research design. CV and AR contributed to the research design. All authors contributed to the article and approved the submitted version.

## Conflict of Interest

The authors declare that the research was conducted in the absence of any commercial or financial relationships that could be construed as a potential conflict of interest.

## Publisher’s Note

All claims expressed in this article are solely those of the authors and do not necessarily represent those of their affiliated organizations, or those of the publisher, the editors and the reviewers. Any product that may be evaluated in this article, or claim that may be made by its manufacturer, is not guaranteed or endorsed by the publisher.

## Footnotes

1 Experimenting, Experiencing, Reflecting (EER) is led by professor Andreas Roepstorff (Aarhus University) and artist Olafur Eliassson, funded by the Carlsberg Foundation Semper Ardens grant (2019–2023).

2 An initial description of the score including pictures can be found at https://www.eer.info/activities/sharing-perspectives-3.

3 This part of the SP score builds on Steve Paxton’s 1975 small dance score (Paxton, 1992).

4 This part of the SP score builds on Karen Barad’s work on touching (Barad, 2012).

5 We preselected these words in order to cover both positive, negative and indifferent experiences.

6 Since the gender of participants has not been relevant to the analysis, we use gender neutral pseudonyms and pronouns (they/them) when referring to participants.

7 See https://www.eer.info/activities/sharing-perspectives-3 for images from the SP score at Tate Modern.

8 The SP score’s interplay with Olafur Eliasson’s work will be discussed in a separate publication.

9 We note that even the participants quoted here responded in the anonymous questionnaire that they found the experience eye-opening and joyful and would recommend it to their friends.
