# Supplementary material for: Sharing Perspectives: Inviting Playful Curiosity Into Museum Spaces Through a Performative Score
Source: Front Psychol. 2022 Jun 9;13:825625. doi: 10.3389/fpsyg.2022.825625 (PMC9218353; doi:10.3389/fpsyg.2022.825625)
Supplement: Supplementary file 1 [file Data_Sheet_1.PDF]

Appendix A: Interview guide for interviews with participants in the SP score

- How did you come to make the decisions of the positions?
- Do you know each other in advance?
- What was it like to take your partner's position?
- Was there anything that surprised you about your partner's position?
- Did you learn anything about your partner?
- Did you learn anything about yourself?
- Were you paying attention to the artworks, your body, the space or other people?
- How did your attention change during the experiment?
- Was the experiment different to how you would normally see an exhibition?
- Was the experience meaningful to you?
- Is there anything you would like to add?
