# Supplementary material for: Sharing Perspectives: Inviting Playful Curiosity Into Museum Spaces Through a Performative Score
Source: Front Psychol. 2022 Jun 9;13:825625. doi: 10.3389/fpsyg.2022.825625 (PMC9218353; doi:10.3389/fpsyg.2022.825625)
Supplement: Supplementary file 2 [file Data_Sheet_2.PDF]

Appendix B: Questionnaire presented to participants in the SP score

1) How often do you visit art galleries? (Choose one of the following)
    a) Once a month or more
    b) 6-10 times a year
    c) 2-5 times a year
    d) Once a year or less
    e) I work in a gallery/museum
2) Have you seen Olafur Eliasson's exhibition at Tate Modern before taking part in today's experiment? (Choose one of the following)
    a) Yes
    b) No
3) Did you know your partner in the experiment in advance? (Choose one of the following)
    a) Never met before
    b) We are recent acquaintances
    c) We are long-term friends/partners/colleagues
4) Do you regularly work with this type of practices (e.g. acting, meditation, contact improvisation, body awareness)? (Choose one of the following)
    a) Once a month or more
    b) 6-10 times a year
    c) 2-5 times a year
    d) Once a year or less
    e) Professional practices
5) Were you surprised by your partner's choice of perspective? (Choose one of the following)
    a) I would have never chosen them myself
    b) One or two of them were surprising
    c) No, they could have been my own
6) Did you learn something about your partner? (Choose one of the following)
    a) Yes
    b) No

    6a) Please explain your response (Free text field)
7) How would you describe the overall experience? (You may choose more than one word)
    a) Demanding
    b) Fast
    c) Annoying
    d) Easy
    e) Trivial
    f) Eye-opening
    g) Slow
    h) Boring
    i) Joyful
8) Exploration of 'Beauty' (Rate from 1–10)
9) Choosing your own position (Rate from 1–10)
10) Sharing your positions with your partner (Rate from 1–10)
11) Exploring your partner's position (Rate from 1–10)
12) Talking about your experiences (Rate from 1–10)
13) Revisiting the positions with a camera (Rate from 1–10)
14) Filling in this questionnaire (Rate from 1–10)
15) Overall experience of Experiment (Rate from 1–10)
16) Would you recommend participation in the experiment to a friend?
    a) Yes
    b) No

    16a) Please explain your response (Free text field)
17) Would you like to give us any other feedback about your experience in this experiment? (Free text field)
