# Supplementary material for: Sharing Perspectives: Inviting Playful Curiosity Into Museum Spaces Through a Performative Score
Source: Front Psychol. 2022 Jun 9;13:825625. doi: 10.3389/fpsyg.2022.825625 (PMC9218353; doi:10.3389/fpsyg.2022.825625)
Supplement: Supplementary file 3 [file Data_Sheet_3.PDF]

**Appendix C: Interview displays**

| Interview display 1 | **Interview 1: EER471 & EER489** | **Interview 2: EER775 & EER263** | **Interview 3: EER998 & EER999** | **Interview 4: EER250 & EER490** |
|---|---|---|---|---|
| **How did you come to make the decisions of the positions?** | Build on intro, getting in to body and relaxing. Walk slowly, follow attention, not restricting myself. Not a lot of reflecting when choosing. Looking for new ways of seeing the work. Keeping an open mind. Interested in relationship between people and artwork. I tended to go to spaces with fewer people. | Get away from other people, trying to pay attention to other things than the visitors, and then slowly opening back up. Finding comfortable places. Instinctive. Finding safe and quiet spaces. | Places that would be interesting for my partner. Places where I could feel the invisible energy of the work. Introduction made me move in slow pace, I flowed with that. Common for my positions was that they were directed towards other people. | Looking for places where I felt comfortable. Places that stimulated me somehow. Looking for interesting spaces, something I hadn'et seen at first. But also to be safe. |
| **Do you know each other in advance?** | No, but both work at Tate. | No. | No. | No. |
| **What was it like to take your partner's position?** | Something I wouldn't have done. Personal angles. Refreshing. Unorthodox. Unsettling. Noticing things I otherwise wouldn't have. | Beautiful. Discovering secrets. Looking at things you wouldn't have thought of yourself. Intimate feeling. Exposed. Intense. Noticing details. | My partner found not normal places that were out of my imaginations. Like a dance. Unexpected positions. Seeing works from new angles. | Wondering why my partner chose this. And then try to feel what my partner feels. Accepting that this must be and interesting position and trying to figure out how. A safe feeling. Feel in a different world. Having everything evolve around me. Wrapped up. Covered up with sound and visuals |
| **Was there anything that surprised you about your partner's position?** | Spaces that I wouldn't have chosen. Trying to focus on what this position is. Nice to be sharing. | Yes. Things I wouldn't have thought about. Connecting more with the artwork. Understanding/feeling new aspects of the work. Aware of you body because of the position. Intimate because of closeness to the works. | My attention was drawn to how the other people interplayed with the work in the crowded space. A treasure to me. Surprised my how my partner physically place their body. New position prioritizes some senses. Hearing the work instead of seeing it. | - |
| **Did you learn anything about your partner?** | - | Showing special places to me. Hard to say I learned something about your personal perspective. Seeing new perspectives. | My partner saw different things than me. Making me connect with the work on a new and more sensory level. Maybe learnt how my partner would move and sense in the exhibit. But because of the lack of | Yes. The small differences in perception. I learned that I could experience the room from the centre. |

**Appendix C: Interview displays**

| | | | | |
|---|---|---|---|---|
| | | | words I didn't learn much about their person. | |
| **Did you learn anything about yourself?** | - | I feel comfortable in the periphery. But suddenly you feel at the centre of the work, or part of the work. The work is activated when you engage with it. | Similar ways of attuning to details. Every moment, touch or sensation is ephemeral, and I tried to seize it. | - |
| **Were you paying attention to artwork, body, space or other people?** | Very conscious of body in relation to space. Later to the other people. To feel disorientated but safe at the same time. | The body. Feeling myself in the space. | Other people and how the engage with work. You're in another space and things are happening around you. Connecting more sensuously to the work. | The space and the people in it. The sounds surrounded me. Finding a place where I could feel the space and the other people. Realizing that things moce when you take time. |
| **How did your attention change during the experiment?** | Feeling like in a bowl of water. Safe. Feeling like in a different speed. Feeling centred. A sensation of being in a slightly different dimension. | Trying to slow down attention, but influenced by other people. Feels like a treat to take 20 minutes to notice yourself. Reconnecting with the body. Attention moving between people, body and work all the time. | "I want to live in a slow pace of life now." Introduction slows you down and changes attention. Everyone else were moving at same pace, and that made me aware of them. Different positions also changes attention. | I became more focussed and centred. After being in the positions for a while I realized how things where shifting around me. To take time and feel. |
| **Was the experiment different to how you would normally see an exhibition?** | - | Yes, approach completely different. Introduction made it more focused. | I would leave if there were so many people, but this was amazing. I never felt this before in an exhibition. The slow pace made me more attentive, very different than normally. | - |
| **Was the experience meaningful to you?** | Yes. Opened my eyes to things I hadn't seen before. | Yes. Good with time to connect, feel and concentrate. Great to pay attention to your own perception. Immersive. | Yes. The intro slowed us down so we had time to feel the exhibition and each other. Learning a new way of moving. | Yes. Developing myself and my work. Beautiful and surprising how we were doing similar and different things at the same time. |
| **Anything you would like to add?** | People were copying us. | Other people also took up out positions. Magical and like a movie. Feeling vibrations of the artwork. | - | - |

**Appendix C: Interview displays**

| Interview display 2 | **Interview 5: EER439 & EER355** | **Interview 6: EER961 & EER226** | **Interview 7: EER311 & EER114** | **Interview 8: EERXXX & EERXXX** |
|---|---|---|---|---|
| **How did you come to make the decisions of the positions?** | Drawn away from people. Looking for safe spaces. Intuitive. Gravitating towards what felt good. Feeling in my own world. Drawn into visuals that overpowering all the people in the room. | Finding yourself in a position rather than choosing. Looking for comfort. Away from people. Sitting, standing. Hand specific space in mind. Conscious of being visible. | Finding interesting new positions to see the artwork. Kneeling down, lower perspectives. Moving around and getting stuck in places. Nice to be looking for something to share. Looking for the mesmerizing and sensory, overwhelming. How the installation works. The small an unexpected that captivates you. | Where I felt comfortable. I didn't want to feel exposed or in the way. I wanted to blend in. A mixture of looking at the artwork and the other people's reactions. |
| **Do you know each other in advance?** | Yes, know each other well. | No. (One works at Tate) | No. | No. (One works at Tate) |
| **What was it like to take your partner's position?** | Interesting how partner was drawn to particular spaces. Entertaining. Refreshing. Something I wouldn't have chosen. Interesting. Confusing. Close up and intimate views of people's expressions. | Interesting.  Skin-to-skin empathy. Knowing that you are visible and inviting a certain kind of watching. Felt performative. When on my own I was just enjoying what was playing out in front of me, but when we together it felt like we were performing. Becoming another layer of the exhibition. I see more when you watch me. | Personal. Connected. Interesting to see things I wouldn't have noticed. Similar but different. Felt lucky that somebody is showing me this. | Interesting. Positions I wouldn't have chosen. Good experience to try another's perspective. |
| **Was there anything that surprised you about your partner's position?** | I never thought to stand in their positions. | That I could find calmness in such a busy space. The introduction did that. | Not surprising intellectually, but in reality I was. Learning that may perspective was not the whole perspective. I got humbled. | Yes, because my partner could move more freely than me, because I have my uniform on. |
| **Did you learn anything about your partner?** | Yes. About their physical experience. The way they hold their body. Reminding me that we perceive things completely different. | Yes. Partner retreating to find quiet spaces, which is different from me. Because of my job here I feel watched and witnessed. | A gentle way of perceiving. Felt very personal. Surprised that we were similar, but at the same time different. Enriching each other by sharing. | Different. Sitting down, which made me aware of the movement of the other people. Fascinating why people behave in certain ways, why they feel comfortable. Nice to do with a strange, a different way of meeting someone that is more bodily. |

**Appendix C: Interview displays**

| | | | | |
|---|---|---|---|---|
| **Did you learn anything about yourself?** | I wanted to see how my partner reacted. The practice was familiar to me. Everything is changing, and so are different experiences. | I'm quite forward and calm. I'm attracted to light. Engaging more with the work. Feeling connected to the elements. Each time I share it increases my ability to see, which shows me my inability to see. | That my perspective only is one perspective. You feel less isolated and alone. An embodied experience, rather than just being in the mind. Felt less restricted in movements. Felt allowed to do unusual things. | I don't like to get in the way, which seemed different from my partner. |
| **Were you paying attention to artwork, body, space or other people?** | I felt like I was part of the exhibition. People were looking at us. Felt like actors. People copying us. More aware of the environment. Observing other people. | The others because they were everywhere. And the art is so much about space. Moments where I forgot where I was. A disorientating sense of embodiment. | A lot to the other people and their interaction with the artworks. Aware of my body, but more of the other people. Everyone in one space. An interactive installation. | All of it together. I was more focussed on the work, but also the other people in the space. |
| **How did your attention change during the experiment?** | I got calmer as the experiment went on. Got used to the surroundings and space. Meditative and more attentive. | It increased and softened. Ebbed and flowed. | In the beginning I was orientating myself, than into the groove of the artwork, and then to other people. Felt dazed. A combination of being alone and being social. You find something for yourself but also to share. | In the beginning I tried to remember the instructions, later it got easier. It was intense with so many people. |
| **Was the experiment different to how you would normally see an exhibition?** | Yes. Interesting perspective. Similar to what I would normally do, but some parts different. Like sitting on floor. | - | Yes. Normally I don't get to share. Usually I would be talking with others. This is richer. You build opinion and the break it down. | Yes. It's like playing in the galleries. I enjoy bringing people in and then having an exchange. It create more interaction and conversation. Yes very different. Intense show. |
| **Was the experience meaningful to you?** | Yes. Thought provoking. Great to think about different perspectives. | Yes. Not easy. Rewarding to be forced to stay with something. Start to see unexpected things. | Yes. And for others. A way of realizing the richness of diversity. | Yes. Nice to exchange perspectives and focussing on slightly different things. Nice with the meditation intro. |
| **Anything you would like to add?** | - | - | Peopled copied us. It felt like everybody should do this. Simple output that would be good for everyone. Makes it easier to interact and be accepting. Why don't we do it regularly? | - |

**Appendix C: Interview displays**

| Interview display 3 | **Interview 9: EER495 & EER464** | **Interview 10: EER601 & EER713** | **Interview 11: EER707, EER558 & EER617** | **Interview 12: EER288 & EER556** |
|---|---|---|---|---|
| **How did you come to make the decisions of the positions?** | Listening to what my body was telling me. Feeling the artworks, and thinking about there design. Sensing the spaces. Thinking of the space as a whole. | Where I felt the most connected with the work. The intro was foreign to me, and only in my third position I felt most connected. I chose the artwork that was most overwhelming to me. Interested in how people use the artwork, how I could see people through the artwork. | Experience more. Touching artwork. Getting another aspect. Get lost in the trees rather than seeing the woods. Becoming aware of us as visible. Becoming part of the exhibit. Looking for details. Visual thing. Going close to the work. Mild non-verbal used communication to decide | A little arbitrary. Places I thought wouldn't be interesting but turned out being after all. About touch. The steepness of the stairs. The cool air from the aircon Joe got me in touch with the movement of the air. |
| **Do you know each other in advance?** | No. | No. (One works at Tate) | Yes, know each other well. | Yes. Medium well. |
| **What was it like to take your partner's position?** | I tried to find a position and then tried to imagine how you are in your body. Grateful of the beauty you showed me. Changing conception of the artwork. Trying to understand how you bodily felt and getting a closer connection. It felt like a reward. We created a connection. | Thinking about why my partner made their choices. But also trying to feel their feeling. Not feeling comfortable in the same positions. Thinking about another person's body. A new experience for me. Feeling an energy. Things I would never have thought about. Trying to understand your point of view. | Your theatrical walking made me aware of my walking. Negotiation on leading, moving towards symbiosis. Nice to follow someone's way of seeing. Trying to get into the other's mind-set. Realize difference in how holding body. Differences in comfort. | Interesting. Needed trust. Something I wouldn't normally do. Doing things against customs. Violating norms of space. Not just one weirdo. |
| **Was there anything that surprised you about your partner's position?** | Views of the artwork, and ways of engaging with it. Things I hadn't seen before. That there are different perspectives. A communication that's different than with words. | I would have never thought about looking through whole in the spiral view. Slightly out of comfort zone. Breaking barriers of how you are supposed to behave. Felt shy from people looking. | Yours were more sensory. Thought I knew what it was about when seeing the position, but then I didn't. | Yours were about feeling. Yours were about finding new visual experiences. Surprised sitting on floor. |
| **Did you learn anything about your partner?** | It felt like you were very exploratory, and sometimes like we were violating something. Fascinating to see that you had completely different movements. | Powerful because we didn't know each other. Being led and to lead was nice. Connecting without feeling shy or ashamed. It reminded me that If we give it a chance we share more than we think. | Yes. How we experience differently. Engaged in a playful activity and learning where rules are. Becoming aware of people around me, and of restrain. | I learned that you were touch-oriented. I learned that you liked getting a vantage point to observe from the periphery. |
| **Did you learn anything about yourself?** | Felt like I was part of the exhibition on so many levels. Felt like I had created something with my interaction. I didn't want to make a spectacle. | That I care about how people move in the space and in relation to the artwork. How we react. The spiritual connection to the artwork. Feeling art without talking. | Nervous of being the weirdo. I don't like being in the centre. | I only stayed in the centre. I was very visual. Things that are obvious to me are not to other's |

**Appendix C: Interview displays**

| | | | | |
|---|---|---|---|---|
| **Were you paying attention to artwork, body, space or other people?** | All of it. But also shifting in between. | More to the body, maybe because of intro, which made me connect with my body, and feeling aware of other bodies as well. | Everything mixed together. Stopped prioritizing artwork over random bit of floor. One total installation. Becoming blind to the hierarchy of objects and people and interactions. Inspiring new ways of engaging. Looking indirectly at the artwork. | Interaction between artwork and others. How they were restricted in their movements. Listening to others' conversations changed my experience of the artwork. |
| **How did your attention change during the experiment?** | Hard to pin down, all over the place. Overwhelmed in the beginning, but then settled down more. But very focused all the time. Felt like being part of the exhibit. | Interesting when to go out of the positions. We got more comfortable as time went on, and we stayed longer. Attention gravitated towards the artwork throughout experiment, and less on what was happening between us. | In a meditative state. Very slow. Patience increased with time. Noticing all the things that happen as you walk. Slowly find that less is more. Creating new ways of communicating non-verbally. Feeling part of the artwork. | I got into a meditative state with time. Really slowed down. I could sit in that corner all day. |
| **Was the experiment different to how you would normally see an exhibition?** | The only time I've seen an exhibition where I truly connected. I wish I'd done it more often. Breaking down barriers of feeling disconnected from an artist I've never met. | It is revolutionary! Instead of experiencing from an individual point of view. It requires a commitment and a safe space. Inspiring me for my work at Tate. | The show encourages this. Like a playground. As a platform that boosted playfulness. But I think you could explore any exhibition like this. I'll think about it more in the next show I see. | - |
| **Was the experience meaningful to you?** | Yes very much. Galleries can me intimidating, but this broke down barriers. Would be great to do with my family. Opening up sensitivity. Taking your body to the space. I can understand when somebody makes work because it was happening to me. | Yes. | Yes. I want to practice it more consciously next time I'm in a show. Like the booze at a party. Breaking your inhibitions. Smashing through barriers. | Yes. Not deeply, but interesting and cool. Interesting to see how different your things were than my things. I might try this in the future in other exhibits |
| **Is there anything you would like to add?** | Very conceptual. Feeling the body and thinking about what I'm feeling. | People copying us. | Creating new ways to behave as we walked. People copied us. Lost sense of time, but wasn't feeling impatient. Rare moment of relaxation and concentration. Like the intro. As a gentle way of playing, building up confidence and shared connectivity. | You can direct your partner to certain things, but you can't make them notice steepness or cold. |
| | | | | |

**Appendix C: Interview displays**

| Interview display 4 | **Interview 13: EER337 & EER954** | **Interview 14: EER108 & EER636** | **Interview 15: EER809 & EER178** | **Interview 16: EERYYY and EERYYY** |
|---|---|---|---|---|
| **How did you come to make the decisions of the positions?** | Interested in other people and their interaction with the space. Quieter spaces to view the pieces. Look at them from different angles. | What I found interesting and what was doable because of busyness. Looking for an angle showing the artwork and the reactions of people. First thing that came to my eye. Wanting to do something differently each time. Something bodily. An optical thing. What do I see. I let my self be drawn in. | The interaction between the space and the people. Things that were not part of the exhibition, creating a contradiction. Out of luck. Walking around and getting attracted to things. The sound and touch of the artwork. My minded just clicked. | - |
| **Do you know each other in advance?** | Yes. Know each other well. | No. | No. | Yes. Medium well. |
| **What was it like to take your partner's position?** | Partner notices details and lights and patterns. Things I didn't notice or would have missed. More relaxed when in partners positions. Nice to hand over the reins. Able to get lost in the space more. Going into partners positions took me out of myself. | It was very often not what I thought. The physicality of it. Made me see things I hadn't expected or seen otherwise. | Embodying the ice. I had to adjust my posture to experience what he experienced. I didn't like the exhibition, but he made me slow down and create my own spots. Interesting not to talk, you might experience more. Something beyond the verbal. | Very nice. Perspective I hadn't seen before. Not only embodying, but taking the same physical position as the other makes it very different. Really felt like stepping into her perspective. Like stepping out of you comfort zone. Trying to embody her movements. Sometimes that was uncomfortable. |
| **Was there anything that surprised you about your partner's position?** | No. | Yes. A Shift of what was possible to experience. Things that I would not normally be aware of, which influenced my way of looking afterwards. | - | Yes. It took time to realize that the light was moving. Tangibly feeling the wall was different for me. |
| **Did you learn anything about your partner?** | She has an eye for detail. | Hard to know without discussing. | | Makes you know the person differently. Communicating without speaking puts you in a different and more empathetic state of mind. Maybe not with a dramatic issue in their life, but you can see how a person views something. |

**Appendix C: Interview displays**

| | | | | |
|---|---|---|---|---|
| **Did you learn anything about yourself?** | Partner had nice flow. I was more aware of my movement when going to their positions. I realized I was more interested in others' interaction with the work than the work itself. | I felt on show myself, because I'm doing something odd. Normally always on the edge of physically interacting with artworks, but here it's part of it. How much that's influenced by the angle you take. | Yes, that I should break the rules of where you can go. | She was seeing things from the outside, like someone watching the ocean, whereas I would just go into the water. She has a physical approach, whereas I'm more of a visual person. I liked stepping into that more physical role. I'll be paying more attention to the space itself. The way it seems like it was meant to be experienced. Realizing that I've been living fast paced. A meditative experience. |
| **Were you paying attention to artwork, body, space or other people?** | The others' interaction with the works. Intro took you into your body. | Depends. In some artworks people were very involved. All of those things. Nice little interactions between the things. | The other people and their interaction with the space and the work. | The work, but so much is related to the space and people so you can't ignore that. But I would have liked to be less aware of the people and preferred it to be empty. I tried to feel my body, but was also focussed on the space. Observing and then feeling. Sometimes other people added to the experience. |
| **How did your attention change during the experiment?** | More relaxed as time went on. | Difference between finding and sharing. With the intro I was inspired to go close to things, and that allowed me to enter into an internal space excluding others. | It's difficult for me to concentrate on one thing, so my attention jumps. | People were distracting |
| **Was the experiment different to how you would normally see an exhibition?** | More sensorial and non-intellectual. This has given me a different perspective which is different from what I normally do. | Yes. Normally we would speak a lot and that can be disturbing. | No. Maybe I was a little bit more focussed. Could be a new way of seeing artworks together. | Completely different. Instructions were beautiful. More people should do it. Feels more authentic. A different way of interacting with art, because you are |

| | | | | |
|---|---|---|---|---|
| | | | | doing it together and have to be aware of each other. Intro gave me permission to embody. |
| **Was the experience meaningful to you?** | Yes. I would do it again. | Yes. By taking somebody else's posture you might understand them better. I'd like to try it again. Nice not to speak | Not mind blowing, but I got more and more into it. It was good to pair up. | Yes. Mystical. Divine experience. Taking you out of the box. Related to my work and studies. Nice to show some else around. Making you aware of your boundaries and comforts |
| **Anything you would like to add?** | | This is what conceptualists should have done. Engage everybody and breaking down expectations of education. | - | A reminder to sit and experience what you're actually living in. Challenging the ways of experiencing reality. Challenging gallery norms. Makes you feel more connected to the space and artworks. |

**Appendix C: Interview displays**

| Interview display 5 | **Interview 17: EER835 & EER492** |
|---|---|
| **How did you come to make the decisions of the positions?** | Go to the pieces I was drawn to and find the most comfortable position to view it. Challenging the ways of viewing. Balancing comfort with challenge. Avoiding too many people, so I could be immersed. It became easier as time went on. |
| **Do you know each other in advance?** | Yes. Work together at Tate. |
| **What was it like to take your partner's position?** | Slightly strange and challenging. Wearing a uniform affected it, and I had to switch the work mind-set off which took a while. Seeing things I hadn't seen before. The work was able to speak to me in a different language. Not speaking added a different and gentler dimension. |
| **Was there anything that surprised you about your partner's position?** | I had to observe your actual posture. Usually I'm quite self- conscious, but now I wasn't. Seeing things in new ways I hadn't thought about. You made me realize how artworks that I didn't like were actually interesting. |
| **Did you learn anything about your partner?** | They were very reflective in looking at the work. We made almost similar choices. Small changes in positions showed me that we are all similar but different. |
| **Did you learn anything about yourself?** | Almost like an out of body experience. Feeling what she's feeling without talking, because you are physically taking her point of view. Not speaking was powerful. |
| **Were you paying attention to artwork, body, space or other people?** | The intro made me aware of my body, which helped me concentrating on the work but also how you react to the room. It became insular, it made me less distracted by the people. Instead it was seeing them as part of the experience. I tried not to shift to much, but the intro got me into a mindful state, where I could really feel my body. |
| **How did your attention change during the experiment?** | I got more relaxed and focussing got easier. The more time went on I got more and more into what I was doing, my body and how I was reacting to the space. |
| **Was the experiment different to how you would normally see an exhibition?** | Not so much, it's quite related to my work. But it inspires me a lot and I want to incorporate it in my work here, because I see the value of truly being embodied in the space. This really lifted me out of my regular exhibition practice. I would love to do this again in another space. |
| **Was the experience meaningful to you?** | Yes |
| **Anything you would like to add?** | People were copying us, and that was really weird. |
