# Supplementary material for: Sharing Perspectives: Inviting Playful Curiosity Into Museum Spaces Through a Performative Score
Source: Front Psychol. 2022 Jun 9;13:825625. doi: 10.3389/fpsyg.2022.825625 (PMC9218353; doi:10.3389/fpsyg.2022.825625)
Supplement: Supplementary file 4 [file Data_Sheet_4.PDF]

## Appendix D: Code overview

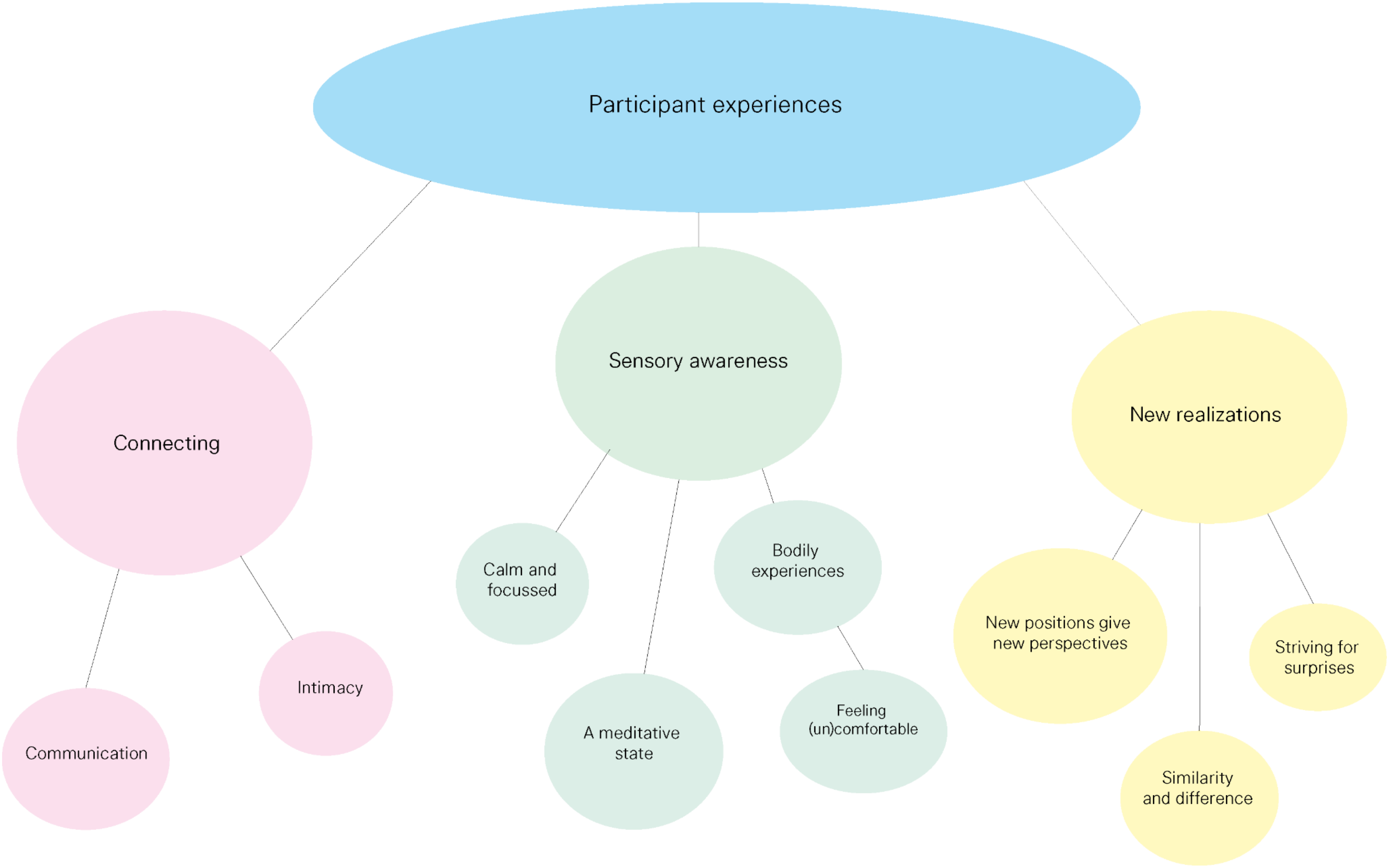

| Code | Code description | Coding example 1 | Coding example 2 |
|---|---|---|---|
| Connecting | Experiences of novel connections to either self, space, artworks or partner | "And you are connecting with that object, and feeling for at and trying to say 'How can I respect you with my actions?' It is a really powerful experience" | "I think I'd take a cup of tea with you now. We didn't know each other, but it's nice to build that relationship with somebody, through their eyes, through their perspective. And it just brings the exhibition more – you're not rushing through, you get the sense of other people going through." |
| Communication | Experiences of novel ways communicating connections | "I think because we weren't speaking, I think that added another dimension as well. I felt quite a gentle kind of way of showing each other how to look at something, rather than being dictated or shown or told. You fell into it way easier." | "It's kind of weirdly skilful, in the sense that it doesn't come naturally and now I think I've got a thing, which I half intended to do, but not so consciously, which I want to now practice more consciously next time I'm in shows. I like the element of not speaking, and of just existing in, and also doing that with a person is really interesting. I'd like to suggest to my friends that we do something similar next time." |
| Intimacy | Feelings of intimacy with partner and/or space | "It was beautiful. It was like discovering secrets somehow. Looking at things that you – I mean the same place, same time, same everything, but looking into things that you wouldn't discover by yourself. Like quickly – it was like somebody telling you a secret, it was that feeling. " | "First of all, standing in silence without speaking, and getting to – so trying to see like that person is seeing, puts you in a different psychological state of mind, in which you can empathize with a person. Maybe you're not empathizing with a dramatic issue in the life of the person, but you can really see how a person views something." |
| Sensory awareness | New attention to sensory capacities | "I felt my partner had a really nice flow as she moved through the exhibition. I think – I didn't pay much attention to how [*I*] got to one space from another, but I think I found I was more aware of my movement, when I was going in to my partner's position. I was more aware of my body, or the way I moved and how slow I was walking, or how that changed the pieces around as well. " | "I think, because yours is more like – it felt more – it's more sensory, or something like touch orientated, and I quite like going from having a light on your face, and it's on the same side, and then going to a cold bronze, and then that kind of contrast between – my face was getting kind of warm." |
| Calm and focussed | Surprised to find calmness in busy space | "I found it surprising that I was able, despite all these people in the space, who are very disturbing, to come to a place of quietness and calm. And I think without this exercise or procedure, I would have probably left the exhibition feeling quite annoyed. Because it's so busy, and you constantly just feel like "Oooh". It's hard to enjoy it, it's really hard to." | "I'm now wearing my uniform, and usually at work, we are taught to pay attention to what's around us. So, the first challenge is to switch off all of those things. And I was wondering if people come to me and say to me 'Oh, can you help me?'. So that was my first concern. But once I switched off that perspective, then I was able to be calm and be more observant And I was seeing things I previously didn't see before. And in fact the works ware able to speak to me in a different language. It wasn't just English, it was an emotional language." |

| | | | |
|---|---|---|---|
| A meditative state | Being transported to different state of mind | "IP1: I think because you're in this kind of strange meditative thing, it's like walking through the galleries, I was even going slower than normal gallery pace. It's crazy slow, but I then think that's the whole part of walking through and then stopping until you find – you're just searching, until that space is the correct one or something.<br><br>IP2: Definitely. I found my patience increased, as time went on. It's like getting slower, and slower, and slower, and it almost could have gone on forever. And as you slowly start to notice all of the different things that you do as you walk, you can start to just get interested in the first muscle that you move. So you can slowly start to find that less, and less, and less, is like more, and more.<br><br>IP1: Yeah. And then speaking again is weird." | "Well, at first I couldn't find anywhere, and I wondered if it would be cool, if after 18 minutes, I said 'I couldn't find anywhere', and I realized it wouldn't. So, I walked around, and then I just tried to find somewhere. So, it was a little bit arbitrary, but even though we weren't supposed to find an interesting place, I found myself looking for an interesting place. So, I pretty much found really interesting places. Places I didn't think would be interesting, but then they were. And then I dropped into this meditative state, so that was cool. |
| Bodily experiences | Experiences connected to the body | "I think receiving the instructions is very beautiful, because it makes you feel the area more deeply. It's like an exercise with the pieces, not just like walking through. Ithink that there is a [status quo] about going to a gallery, you just walk through it, and that's it. You just see it, maybe you don't embody it. And being told to embody it, I think is really fantastic, even there maybe should be like – that should be on the wall, maybe so people do it more often. Because it feels like a more authentic way of feeling the piece. Dedicating it time and different ways of not just seeing it, but sensing it." | "Because my partner is using a cane, so in some of the stances, I was kind of going into his physical embodiment in that moment. And I could feel how that is in your body. Like, holding yourself in that position." |
| Feeling (un)comfortable | Explicit experiences of comfort or discomfort | "I think, other people would have thought that I was in the way or something, because I chose to stand in quite an inconvenient position, but I was so comfortable and relaxed in my visual world, oblivious to everyone else." | "I followed you the first time around, and I tried to mirror your movements, because I tried to understand how you feel in your body. And it was interesting to realize that I can't. Because, when we were squatting down it was uncomfortable for me, but it must have been comfortable for you." |
| New realizations | Experiencing new realizations or revelations | "So I realized while walking through the exhibition, that what I am really interested in is people. So, I am interested in how people use the | "For me a surprise was, we knelt down and looked into the big spiral tunnel from the outside, but through one of the holes. On our knees, slightly uncomfortable and just seeing |

| | | | |
|---|---|---|---|
| | | artwork. So I realized that everything that I found interesting where I stopped, was something about looking at how people get reflected – how I could see people through the artwork, so I think I chose everything because it gave me a new way of seeing people. | time pass and bodies pass, and just sort of seeing that. That was my first feeling of sitting there and looking. "Okay I'm looking at time passing. [I:Mm] I'm looking at people moving and so on." And suddenly, I had this really physical experience of the whole thing is shaking, because of people walking in there. [I: Yeah [Laughs]] So, the whole thing is like breathing, and suddenly it became also a body, and that was a surprise to me. I didn't know. It was like "Oh, the whole thing is moving," you know?" |
| New positions give new perspectives | Shifting physical position in a space changes perspectives and understanding of space | "But I'm also amazed how this kind of communication and the misunderstanding there of course always happens with, with understandings. I'm pretty sure, you look at someone and they look back, and you say 'Yeah, I completely understand what they want to say'. And actually, no you don't. You completely understand the opposite." | "A lot of the time, when I saw you start, I was sort of thinking "Oh, that's nice and simple, ooh that's nice and simple of you". And then I'd eat my words every time, because I'd see how you'd positioned yourself, and once you are there, there's a lot more complexity going on, and you'd arranged stuff, just by positioning. Visually, things were arranged, but you wouldn't know it, looking from the outside. You had to take that position to see what it is that was quite special about how you had arranged the perspective." |
| Similarity and difference | Reflections on the relations between similarity and difference in perspective and perception between partners and positions | "I was surprised that the feeling I got, was that we all are interested in kind of similar things, so we are not so different. But at the same time we're different, because we instantiate that interest in a different way. So, we can really enrich each other with showing this. And the feeling I got also, to some extent, is feels opposite from what I see in social media, where you just get your own perspective reinforced. Here it was a bit the opposite, and it felt like everybody should do this. | "IP: I think for me it's interesting that she made similar choices as I did, almost similar viewpoints actually. I mean, it's just slightly different ways of looking, but it was pretty much similar.<br><br>I: And did it make a difference? Those small changes between the way you were standing, and the way she would be standing, did that tell you anything about her?<br><br>IP1: I think we're all individuals, aren't we? And I think learning to appreciate the individuality in our commonness is so powerful. Just appreciate – and I can even respect that. So, it might be similar, but it's different, it's unique and individual." |
| Striving for surprises | An explicit urge to find surprising and/or new perspectives | "My spaces – it's interesting, I wanted them to be interesting, so I wanted them to show me something that I hadn't seen at first, and then I realized that I also wanted to feel safe and protected." | "For me, I just tried to find the most interesting position for myself, and how I would perceive the object or the installation. So, I went for example down on a lower level and I knelt down to see some lights for example, and not look at it from up here, but from a lower perspective." |
